# Chemical genetics and strigolactone perception

**DOI:** 10.12688/f1000research.11379.1

**Published:** 2017-06-22

**Authors:** Shelley Lumba, Michael Bunsick, Peter McCourt

**Affiliations:** 1 Cell and Systems Biology, University of Toronto, and the Centre for the Analysis of Genome Evolution and Function, University of Toronto, Toronto, ON, M5S 3B2, Canada

**Keywords:** chemical biology, plant development, hormones, signal transduction, Arabidopsis, Striga, parasitic plants

## Abstract

Strigolactones (SLs) are a collection of related small molecules that act as hormones in plant growth and development. Intriguingly, SLs also act as ecological communicators between plants and mycorrhizal fungi and between host plants and a collection of parasitic plant species. In the case of mycorrhizal fungi, SLs exude into the soil from host roots to attract fungal hyphae for a beneficial interaction. In the case of parasitic plants, however, root-exuded SLs cause dormant parasitic plant seeds to germinate, thereby allowing the resulting seedling to infect the host and withdraw nutrients. Because a laboratory-friendly model does not exist for parasitic plants, researchers are currently using information gleaned from model plants like *Arabidopsis* in combination with the chemical probes developed through chemical genetics to understand SL perception of parasitic plants. This work first shows that understanding SL signaling is useful in developing chemical probes that perturb SL perception. Second, it indicates that the chemical space available to probe SL signaling in both model and parasitic plants is sizeable. Because these parasitic pests represent a major concern for food insecurity in the developing world, there is great need for chemical approaches to uncover novel lead compounds that perturb parasitic plant infections.

## Introduction

Small organic molecules are important sources of signaling hormones in both plants and animals ^1– 3^. With respect to plants, around ten small molecule hormones have been identified so far, and core signaling pathways for each of these hormones have been characterized ^3^. Much of the success in understanding how small molecule hormones are perceived in plants comes from genetic analysis, which usually involves finding mutants with altered hormone sensitivity followed by molecular identification of the wild-type protein involved. The power of genetics in dissecting plant hormone signaling was impressive, especially since many players in plant hormone signaling were genetically redundant, which precluded their identification as simple recessive mutations ^4– 6^. Many saturated genetic screens, however, led to the identification of rare dominant mutations ^7– 9^ which served as toeholds in building many signaling pathways. The subsequent development of well-characterized mutant knockout collections in *Arabidopsis* ^10^ allowed more components to be validated through the construction of multiple loss-of-function mutant lines in redundant steps.

The contribution of genetics to unravelling plant hormone biology is self-evident, which raises interest in where genetic analysis can make future contributions. A good example of the evolution of genetic approaches in plant hormone signaling is now occurring in the field of chemical genetics ^11^. Simply defined, chemical genetics involves the development of chemical agonists and antagonists to probe biological processes. This approach by definition should be well suited for plant hormone signaling since plant hormones are small molecules and as such their receptors should be “druggable” ^12^. However, geneticists often look suspiciously on chemical perturbation experiments because of concerns of off-target effects. Chemical geneticists have tried to address this criticism by defining criteria for what makes a good chemical probe ^13, 14^ ( Table 1). From a sceptic’s perspective, two of these conditions go far to assuage their chemical fears. First, chemical addition to wild-type organisms must clearly and specifically mimic a well-characterized mutant phenotype. For example, the addition of silver ions or 1-methylcyclopropene, both of which antagonize ethylene receptors, results in dark-grown seedlings that look phenotypically similar to mutations that decrease ethylene perception ^15^. A second and more powerful criterion that monitors off-target effects is the use of a “decoder strategy” ^16^. In this case, application of the compound in question to a loss-of-function mutant in the target gene should result in loss of the global gene expression signature generated by compound treatment of the wild-type. For example, addition of the histidine biosynthetic inhibitor 5-amino triazole (5-AT) to a mutant lacking the 5-AT target ( *histidine3* [ *his3*]) resulted in dramatic reductions in the global gene expression signature observed in 5-AT-treated wild-type yeast cells ^16^.

**Table 1.** Some criteria used for determining the utility of a chemical probe.

Chemistry	
Structure	Defined structure
Stability	Stable in test media
Potency	
Biochemical	<100 nM in *in vitro* biochemical assay
Cellular	<1–10 mM in cell or whole organism assay
Analogs	Closely related structures have similar activity
Selectivity	
Inactive analogs	Analogs with no biochemical activity have no biological activity
Genetic	Chemical closely mimics mutant phenotypes
Target	Chemical follows “decoder” parameters ^16^

In plants, perhaps the best example of chemical probe development involved the identification of pyrabactin as a selective agonist of the receptor for the hormone abscisic acid (ABA) ^17^. Pyrabactin was first identified as a general germination inhibitor in a chemical screen in *Arabidopsis*, but its specific role in ABA signaling was suggested by the ability of ABA-insensitive mutants to germinate on pyrabactin. Second, the pyrabactin analog, apyrabactin, showed no biological activity. Finally, although a decoder approach was not applied, global gene expression experiments between seeds treated with ABA or pyrabactin were highly correlated, suggesting few off-target effects.

The identification of pyrabactin allowed the development of genetic screens to identify mutations in an essential gene encoding an ABA receptor that is involved in germination ^17^. Because pyrabactin was a selective ABA agonist, it activated only a subset of ABA receptors and particularly the major one involved in germination ^17^. Thus, resistant mutants to pyrabactin circumvent genetic redundancy issues that cannot be resolved by traditional ABA screens. This result showed how a new approach like chemical genetics, which can identify more selective compounds, can uncover novelty when wedded to an old approach like a traditional forward genetics screen. The pyrabactin story also demonstrated how the identification of a specific chemical probe can have broader applications beyond basic biology. Information on the pyrabactin structure has led to the identification of chemical analogs that could be used to protect important agronomic crops from drought, a process that is mediated by ABA signaling ^18, 19^.

This development of chemicals as both probes for plant hormone signaling and translational leads is clearly exciting. Perhaps there is not a more obvious application of chemicals than in the study of a recently identified collection of chemically related hormones called strigolactones (SLs) ^20, 21^. SLs, like all small molecule plant hormones, have many roles in plant growth ranging from filament growth in nonvascular plants ^22^ to shoot branching ^23^ and root development ^24^ in vascular plants. However, unlike most plant hormones, SLs also have ecological signaling roles. SLs are exuded from plant roots into the rhizosphere, where they attract arbuscular mycorrhizal (AM) fungi for a beneficial interaction that brings water and nutrients to the plant ^25^. Unfortunately, root-exuded SLs also act as a cue to tell a number of parasitic plant species that a host is nearby ^26^. In these cases, obligate parasites of the *Striga*, *Phelipanche*, and *Orobanche* genera have evolved strong seed dormancy, which is broken only when they sense host-derived SLs. After germination, the parasite infects the host with devastating consequences. The range of infestations by the *Striga* genera alone includes much of sub-Saharan Africa, and these infestations are considered to be a major impediment to poverty alleviation on the continent ^27, 28^. Thus, the identification of chemicals that probe SL perception not only will help to understand SL biology but also may lead to the development of new compounds to combat parasitic plant infestations in the developing world.

## SL chemistry and signaling

**Figure 1.**
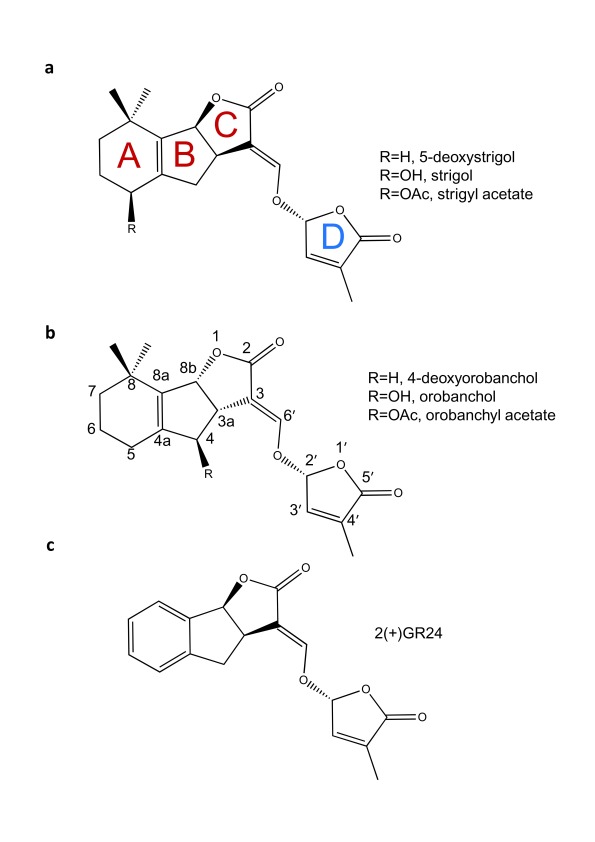
Chemical structure of strigolactone (SL). The chemical structures of naturally occurring SLs can be divided into two families, the orobancol family ( **a**) and the strigol family ( **b**) based on stereochemistry around the BC ring. Chemical differences within a family are related to substitutions (R) on the A or C rings. All naturally occurring SLs found to date have C2'-(R) stereochemistry via the enol-ether bridge that connects the C and D rings. GR24 ( **c**) shown in the C2'-(R) conformation is the most commonly used synthetic SL.

The application of chemical genetics to understanding and perturbing SL functions in parasitic plants requires a basic understanding of both SL chemistry and signaling. The canonical structure of a SL molecule is usually represented as a butenolide ring (D ring) connected to a tricyclic lactone (ABC rings) via an enol-ether bridge ( Figure 1) ^29, 30^. The ABC rings do show natural chemical variation, and to date approximately 20 SLs have now been identified ^29, 30^. SLs can be further categorized based on stereochemistry around the B and C ring into the strigol and orobanchol families ( Figure 1). Finally, stereochemistry at the C2' position appears to be important, with the natural R-isomer showing the best SL activity ^31– 34^.

**Figure 2.**
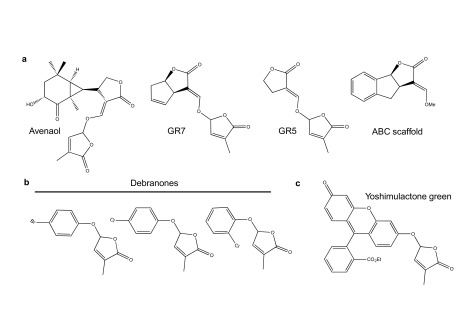
Chemical structures of some strigolactone (SL) agonists with butenolide rings. ( **a**) Each of these SL agonists has an attached butenolide and is expected to be hydrolyzed by D14-type hydrolases. ( **b**) These debranones are three monohalogenated derivatives. The chlorinated derivative on the right is moderately active in *Striga hermonthica* germination assays. ( **c**) Yoshimulactone green (YLG) is hydrolyzed by SL receptors to yield a D ring and fluorescein that fluoresces green.

Further hints as to what parts of a SL molecule are important for perception have come from both natural and synthetic sources ^35^. Avenaol, for example, which is found in root exudates from *Avena strigosa* (black oat) and shows SL activity, has the C and D moieties but lacks a B ring and has an additional carbon between the A and C rings ( Figure 2) ^36^. Synthetic compounds, such as GR7 and GR5, that lack A and B rings but retain CD ring chemistry also show activity in both parasitic and nonparasitic plants, whilst compounds that contain the ABC rings but lack a D ring are inactive ( Figure 2) ^33– 35^. Together, these chemical variants suggest that the C and D rings are essential for SL activity.

**Figure 3.**
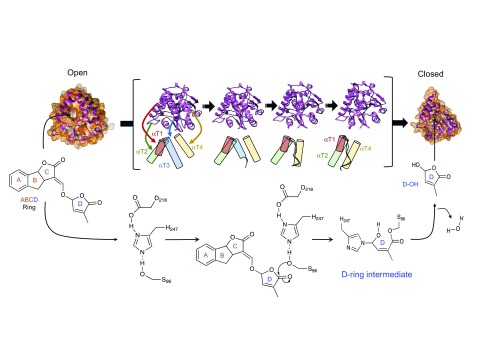
A model of strigolactone (SL) perception. A D14-type receptor without SL is in an unbound open conformation (open). Upon SL binding, the SL is hydrolyzed, releasing the ABC rings. Hydrolysis occurs via a nucleophilic attack by the S96 amino acid of the catalytic triad, releasing an ABC ring. A covalent bond then occurs between the C5' moiety of the D ring and H247, leading to a D-ring intermediate. During this process, it is thought that D14 signaling partners are recruited, which promotes transition of the receptor to a closed state (closed). Receptor transition from an open to a closed configuration is represented by models of four intermediate crystal structures (pink, within the brackets). Below each crystal is a tube representation of the four α helices (αT1–αT4) that form a lid and their positions during the transition from an open to a closed state. As the receptor transitions, the αT1 (brown), αT2 (green), and αT4 (yellow) helices move to close the lid. The αT2 helix becomes an unordered ribbon (blue). A movie of the transition from the open to the closed form can be found in Supplementary File 1. The exact order of events after SL hydrolysis with respect to conformational changes and recruitment of signaling partners remains to be clarified. Based on *in vitro* analysis, it is unclear whether the D ring is irreversibly trapped within the closed receptor. The open structure has the PDB code 4IH4. The closed structure has the PDB code 5HZG.

The enol-ether linkage between the C and D rings also seems to be important. SL receptors in model plants, collectively called D14-type SL receptors after the rice DWARF14 (D14) receptor ^37^, metabolize SLs to generate tricyclic ABC and D ring moieties ^38^. Structurally, D14-type receptors have a canonical α/β-fold consisting of four β-sheets bound by a collection of four α-helices (αT1–αT4), which form a lid encompassing the SL ligand-binding pocket ( Figure 3) ^38– 42^. Within the pocket, there is a functional serine–histidine–aspartate catalytic triad that is required for SL hydrolysis ^38, 41^. Although the order of signaling events after an SL binds a D14-type receptor still needs to be clarified, one simple path appears to be emerging ( Figure 3). As SL enters the ligand-binding pocket, a catalytic serine (Ser96 in the D14 protein from rice) attacks the C5' position of the D ring, resulting in cleavage and release of the tricyclic ABC ring ^43, 44^. The remaining D-ring moiety covalently bonds with the catalytic histidine (His247). This event appears to result in small conformational changes that allow the recruitment of other SL signaling partners. The recruitment of these signaling partners in turn appears to cause a larger conformational change of the receptor to a closed state by trapping either a D-ring intermediate or the D ring itself ^43^. Although many details on timing and order need to be worked out, the covalent bond between the D-ring moiety and D14-type receptors suggests that the metabolism of the SL ligand may be integral to perception and downstream SL signaling ^44^. This also explains why D14-type receptors retain a canonical α/β hydrolase amino acid catalytic triad that shows very slow kinetics of hydrolysis ^38, 41, 44^.

So what about parasitic plant SL signaling? Unlike model plants, D14-type receptors do not appear to be the major player in the germination response of parasites to host-derived SLs. This function falls to a related D14 α/β hydrolase given the name HYPOSENSITIVE TO LIGHT/KARRIKIN INSENSITIVE2 (HTL/KAI2) ^45^. The double-barreled name of HTL/KAI2 is because of two groups independently identifying loss-of-function mutations in this gene based on two phenotypes: 1) hyposensitivity to light (HTL) ^46^ and 2) insensitivity to the smoke-derived germination stimulant karrikin (KAI2) ^47^. The identification of HTL/KAI2 hydrolases as SL receptors in parasitic plants was surprising, as these proteins in model systems do not respond well to naturally occurring C2'-(R) SL isomers and at this time their natural ligand is unknown ^48^. Analysis of *HTL/KAI2* genes from *Striga hermonthica* ( *ShHTL/KAI2*) revealed that these receptors have a range of sensitivity and specificity with respect to the SLs they recognize ^49, 50^. Functional analysis in *Arabidopsis* demonstrated that one receptor, *ShHTL7*, was sufficient to increase the sensitivity of the SL response in *Arabidopsis* to picomolar levels, which is within the concentration range observed for the response of *Striga hermonthica* seed to SLs ^50^. Recent biochemical analysis of ShHTL7 suggested that it may have a similar mode of action to D14-type receptors with respect to transducing an SL signal ^51^. Although a mechanistic understanding of how parasitic plant receptors attain high levels of SL sensitivity has not been clearly elucidated, it does appear that parasitic plant *HTL/KAI2* genes, unlike their nonparasitic plant counterparts, have evolved away from perceiving karrikins to sensing SLs ^45, 50^.

## Chemical screening for SL agonists

The requirement of host-derived SLs for the germination of parasitic plant seed has been a major focus on which to develop strategies to combat these pests ^52, 53^. The logic involves synthesizing cheap SL analogs that are stable in the soil which would germinate parasitic seed in the absence of a host. These compounds are generically called “suicide germination compounds” because these obligate parasites like *Striga hermonthica* will die after germination without a host. The first synthesized suicide germination compounds such as the popular derivative GR24 ( Figure 1) were designed around the SL core structure, but it soon became clear that the chemical space for SL activity was not limited to canonical SL scaffolds. Partially this occurred as hormone-based SL bioassays became routine ^35^. For example, rice ^54^ and pea ^34^ researchers used rescue of SL-deficient branching phenotypes as a screening tool to determine SL activity of their compounds. This approach led to the identification of phenoxy furanone derivatives called debranones (de-branching furanones) that showed good SL activity although they lacked the ABC ring structures and any enol-ether linkages ( Figure 2). This observation that enol-ether chemistry was not required for activity was in part responsible in the development of on-off fluorescent probes such as yoshimulactone green (YLG) that were instrumental in understanding the SL responses in parasitic plants like *Striga hermonthica* ( Figure 2) ^49^. Interestingly, although YLG acts as a SL in both parasitic plant germination and nonparasitic plant branching, debranone was not potent in parasitic plant germination assays ^54, 55^. However, specific chlorine additions on the phenyl ring of the debranone scaffold ( Figure 2) do improve the response of parasitic seeds ^55^. This implies that compounds can be developed that preferentially target the pests without influencing host behavior.

**Figure 4.**
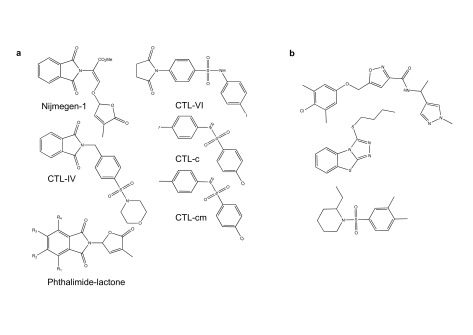
Chemical structures of some strigolactone (SL) agonists lacking butenolide rings. ( **a**) Nijmegen-1 (upper left corner), which contains a butenolide ring, is shown as a reference phthalimide-based SL mimic. The phthalimide lactone core structure (lower left corner) was used to develop new parasitic plant germination stimulants. R-groups represent different places on the core structure where modifications were made. ( **b**) The structures of these three compounds were found by screening for compounds that encourage protein–protein interactions between AtHTL/KAI2 and its F-box partner protein MAX2 ^38^.

This idea of using biology rather than chemistry to guide SL activity has greatly expanded as good large annotated chemical libraries became commercially available ^11^. For example, a chemical screen designed strictly around *Arabidopsi*s germination and early seedling growth identified a collection of succinimide and phthalimide compounds that appeared to impinge on SL biology ^56^. Collectively called cotylimides (CTLs), a number of these compounds have subsequently been shown to bind and activate AtHTL/KAI2 ^57^. The phthalimide structure in some cotylimides (CTL-IV) is also found in Nijmegen-1, a potent SL mimic ^58^. However, unlike Nijmegen-1, CTL compounds do not contain a D ring and certainly are not a hydrolysable substrate. Interestingly, other compounds built on phthalimide lactone scaffolds but lacking enol-ether linkages have good selectivity activity on various *Orobanche* and *Philibanche* species ^59^. Finally, a screen for novel germination agonists developed specifically around the activation of the AtHTL/KAI2 receptor with one of its protein partners, MORE AXILLARY GROWTH2 (MAX2), identified more structurally unrelated compounds that have activity in *Striga hermonthica* germination assays ( Figure 4) ^57^. Thus, biologically based screens appear to be uncovering a myriad of chemical compounds that show little chemical relatedness to canonical SLs. The diversity of compounds with SL activity is surprising, as early work suggested that CD rings and stereochemistry are vital to the SL response. Furthermore, all the known SL receptors have a conserved catalytic triad, indicating that hydrolysis is essential to signaling. Possibly, these new SL agonists bind SL receptors in different places than the canonical SLs and as such do not require hydrolysis for activity. Alternatively, it would also be interesting to test these compounds on catalytically dead tirad mutants. Whatever the case, structural studies with these different compounds will be needed to resolve these issues.

## Chemical screening for SL antagonists

**Figure 5.**
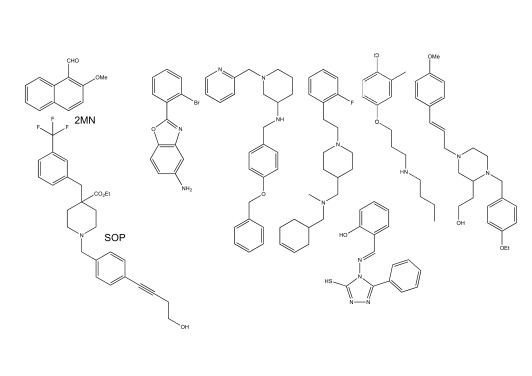
Chemical structures of some strigolactone (SL) antagonists. Within these antagonists, only 2-methoxy-1-naphthaldehyde (2-MN) and soporidine (SOP) have been closely characterized biochemically in model systems and with respect to *Striga hermonthica* germination.

Although chemical genetic screens for new SL agonists are viewed through the prism of SL chemistry, screening for SL antagonists has not had this bias. One of the first attempts to identify SL antagonists involved taking advantage of D14-type receptor structural biology. Compounds were first screened *in silico* for chemicals that would theoretically fit the binding pocket of the rice D14 receptor ^60^. Positive hits were next tested using a yeast two-hybrid assay to find which compounds reduced SL-dependent D14-protein-protein interactions. This approach identified 2-methoxy-1-naphthaldehyde (2-MN) ( Figure 5), and subsequent experiments showed 2-MN interfered with a number of SL-dependent processes in rice and *Arabidopsis* ^60^. 2-MN showed some activity in inhibiting *Striga hermonthica* seed germination, and this reduced potency may reflect the experimental design that was based on D14-type receptors rather than parasitic plant HTL/KAI2 receptors. A crystal structure of the SL receptor (ShHTL5) from *Striga hermonthica* now exists ^50^, and it would be interesting to see how *in silico* screening of this receptor may influence lead compound identification.

Recently, a second SL antagonist screen has been performed using whole plant SL-dependent assays in *Arabidopsis* rather than knowledge of the receptor structure *a priori*. This assay was based on the observation that SLs inhibited *Arabidopsis* hypocotyl elongation. Another advantage to using *Arabidopsis* was a genotype whose hypocotyl was sensitized to SL inhibition, which was used in the primary screen to identify compounds that interfered with SL-dependent inhibition of hypocotyl growth ^61^. Seven compounds were found, one of which was a piperidine-based molecule called soporidine (SOP). In addition to binding AtHTL/KAI2, SOP bound the *Striga hermonthica* receptor ShHTL7 and inhibited its hydrolytic activity. Functionally, SOP inhibited the germination of *Striga hermonthica* seed in the presence of SL ^61^. Thus, hypocotyl-based assays in *Arabidopsis* appear to be a good screening platform to identify lead compounds that may perturb *Striga* germination. None of the antagonists identified so far have significant structural similarity to known naturally occurring SLs. Similar to the case of SL agonists, it is possible that these antagonists bind somewhere else on the receptor to inhibit activity. This again begs for more studies on the structure–function relationships between the SL receptors and the plethora of compounds identified from chemical screens.

## Concluding remarks

The rapid increase in our knowledge of the fundamental biology of SL signaling based on studies on model plants is allowing researchers to test the mechanism by which SLs are perceived in parasitic plants. Furthermore, this information is currently being used to identify new chemical probes that perturb SL perception in both nonparasitic and parasitic plants. Although it is not surprising that small molecules resembling SLs have activity in SL-based plant assays, the identification of a large number of synthetic lead compounds from chemical screens that do not possess canonical SL structures is intriguing. This could simply reflect different binding sites and modes of action. It could also mean that SL receptors, whether from parasitic or nonparasitic plants, are more promiscuous than previously thought with respect to small molecule activation, particularly at higher concentrations.

Biologists are usually taught that compounds that bind a receptor with high affinity are most likely the most relevant *in vivo.* This explanation, however, has never really explained functional low-affinity ligands *in vivo* and many times these compounds are written off as nonspecific and biologically irrelevant. In some sense, this hand-waving argument is akin to early beliefs about “sticky proteins” found in large-scale protein–protein interaction networks. Once thought to be biochemical artifacts, most of these proteins are now viewed as essential components in scale-free signaling networks ^62, 63^. It is now becoming clear from evolutionary studies on small molecule hormone receptors from animals that many small molecule receptors were initially low-affinity sensors for a range of metabolites and later evolved to become high-affinity receptors of particular chemicals ^64– 66^. These models are consistent with suggestions that the ancient HTL/KAI2 receptors may have had reduced chemical specificity that later evolved into high-specificity SL receptors ^67^. Such a model may explain why HTL/KAI2-type receptors were selected over D14-type receptors by parasitic plants, since these species must be able to readily evolve to new host SL ligands and compositions in order to move to new hosts ^68^.

The lack of high-affinity agonists and antagonists could suggest chemical genetics will not yield useful probes and interesting insights. However, although traditionally searches for drugs have been based on finding high-affinity compounds, the pharmaceutical industry has changed its strategy to perform “fragment-based screening”, which is designed to find low-molecular-weight compounds that work in the high micromolar range initially ^69^. Once compounds have been identified, the methods of medicinal chemistry can be used to increase their potency orders of magnitude. In this scenario, lead compounds identified with SL activity would serve as chemical scaffolds for the development of more potent compounds. Applying the same logic, perhaps the addition of D-ring structures to leads implicated in SL function, would increase their efficacy.

Finally, insights into plant versus animal hormone signaling have come from different approaches. Animal cell culture systems ^2^, which are well defined developmentally and easy to handle experimentally, were instrumental in dissecting how small molecule hormones are perceived and signal. By contrast, plant hormone signaling was led by phenotypic screening typically on whole organisms such as *Arabidopsis* ^70^. Chemical genetic analysis of hormone signaling in these two kingdoms will most likely follow the same route, and animal researchers are now more frequently using phenotypic screening as an effective method of drug discovery ^71^. In plant systems, compounds will continue to be identified through some phenotypic screen and the resulting compounds will be the basis for mutational analysis to further understand the mode of action as was seen with pyrabactin ^17^. The grounding of plant chemical genetics in phenotypic screening bodes well for new insights, since phenotypic screening is gathering momentum as a way to re-energize animal drug research ^71, 72^. In this sense, plant biologists are already there.

## Supplementary material

**Supplementary File 1: Changes in SL receptor structure upon SL binding.** A movie of the dynamic change in the Arabidopsis D14 (AtD14) SL receptor conformation upon binding of SL as shown in Figure 2. In its open form (Open) the receptor has an accessible binding pocket to accommodate an SL molecule. Upon binding the receptor begins conformational change that attracts singling partners resulting in a closed form (Closed) that traps a D-ring intermediate within the receptor (see Figure 2). In this movie only the receptor is shown (purple).
